# Human pluripotent stem cell derived midbrain* PITX3^eGFP/w^* neurons: a versatile tool for pharmacological screening and neurodegenerative modeling

**DOI:** 10.3389/fncel.2015.00104

**Published:** 2015-03-31

**Authors:** Bradley Watmuff, Brigham J. Hartley, Cameron P. J. Hunt, Stewart A. Fabb, Colin W. Pouton, John M. Haynes

**Affiliations:** Stem Cell Biology Group, Drug Discovery Biology, Monash Institute of Pharmaceutical Sciences, Monash UniversityParkville, VIC, Australia

**Keywords:** *in vitro* neurodegenerative modeling, human embryonic stem cells, functional characterization, midbrain dopaminergic neurons

## Abstract

*PITX3* expression is confined to adult midbrain dopaminergic (mDA) neurons. In this study we describe the generation and basic functional characteristics of mDA neurons derived from a human pluripotent stem cell (hPSC) line expressing eGFP under the control of the *PITX3* promoter. Flow cytometry showed that eGFP was evident in 15% of the neuron population at day 12 of differentiation and this level was maintained until at least day 80. From days 20 to 80 of differentiation intracellular chloride decreased and throughout this period around ∼20% of *PITX3^eGFP/w^* neurons exhibited spontaneous Ca^2+^ transients (from 3.3 ± 0.3 to 5.0 ± 0.1 min^-1^, respectively). These neurons also responded to any of ATP, glutamate, acetylcholine, or noradrenaline with elevations of intracellular calcium. As neuronal cultures matured more dopamine was released and single *PITX3^eGFP/w^* neurons began to respond to more than one neurotransmitter. MPP^+^ and tumor necrosis factor (TNF), but not prostaglandin E_2_, caused death of the ∼50% of *PITX3^eGFP/w^* neurons (day 80). Tracking eGFP using time lapse confocal microscopy over 24 h demonstrated significant TNF-mediated neurite retraction over time. This work now shows that these *PITX3^eGFP/w^* neurons are amenable to flow cytometry, release dopamine and respond to multiple neurotransmitters with elevations of intracellular calcium, we believe that they represent a versatile system for neuropharmacological and neurotoxicological studies.

## Introduction

Since Parkinson’s disease (PD) has no cure current therapeutics target motor systems to either increase dopamine production or adjust the impact of other neurotransmitters, but these treatments are likely to eventually fail. Human pluripotent stem cell (hPSC) derived midbrain dopaminergic (mDA) neurons have captured the imagination of public and scientists alike as a source of cells for disease arresting cell replacement therapies for PD (Kim et al., 2002; Lindvall et al., 2004). However, our contention is that hPSC-derived mDA neurons, with genetic modifications designed to enhance their utility, could promote the next generation of *in vitro* pathophysiological modeling and drug discovery studies. Recent modeling studies have used reprogramming technology to generate hPSCs derived mDA neurons from patients with PD with an aim to specifically investigate the role of single gene mutations on neuron function and survival (Devine et al., 2011; Nguyen et al., 2011; Sanchez-Danes et al., 2012). More generic model systems often employ neurotoxins such as 6-hydroxydopamine or 1-methyl-4-phenyl-1,2,3,6-tetrahydropyridine (MPTP) to induce degeneration of the A9 dopaminergic neurons. In these models of PD the neurotoxin-induced degeneration of the A9 neurons appears to be dependent upon the activity of tumor necrosis factor alpha (TNF) which is elevated in the striatal tissue of mice and rats following the injection of MPTP or 6-hydroxydopamine (Mogi et al., 1999; Sriram et al., 2002). Critically, suppression of TNF reduces neurodegeneration in these model systems (McCoy et al., 2006, 2008; Sriram et al., 2006). While the underlying causes of PD are the subject of much speculation, this evidence of a role for TNF in the degenerative process is consistent with oxidative stress contributing to PD. In this system a transient initiating factor (an infection or neurotoxin) triggers a chronic cycle of neuroinflammation which promotes clustering of activated microglia in the basal ganglia and striatum of patients with PD, promoting degeneration (Gerhard et al., 2006). Microglia are important regulators of neuron function. In addition to phagocytic activity, they are capable of releasing trophic factors, such as brain cell line-derived neurotrophic factor (BDNF; Nagatsu and Sawada, 2007), as well as a number of pro-inflammatory mediators, including chemokines, reactive oxygen and nitrogen species, prostaglandin E_2_ and TNF. Both TNF and TNF receptor (R1) are elevated in the cerebrospinal fluid and nigral tissue of patients with PD (Mogi et al., 1994, 2000). Also, patients with gain of function mutations in the TNF promoter region show an increased risk of early onset PD (Nishimura et al., 2001; Bialecka et al., 2008). In spite of this evidence there is no direct empirical evidence linking TNF with the death of human A9 neurons.

One of the major problems underlying mechanistic investigations of the role of TNF in human neurodegeneration has been the lack of an adequate *in vitro* system to facilitate these investigations. Human stem cell cultures and methods to push neurons toward a dopaminergic neuron fate have improved considerably over the last 6–7 years. However, a major problem with hPSC-derived mDA neural cultures is that current differentiation protocols, even the few that can be replicated in other laboratories (such as Kriks et al., 2011), still generate a heterogeneous mix of cells in culture that make it difficult to observe or quantify effects. To address this shortcoming we have created a human homologous recombinant stem cell line that expresses a fluorescent reporter gene (eGFP) under the control of a midbrain specific DA neural endogenous promoter (PITX3) to enable the identification of PITX3-eGFP positive (*PITX3^eGFP/w^*) mDA neurons within the culture. We now report that *PITX3^eGFP/w^* neurons are evident from day 20 of differentiation although they take approximately 70 days to reach functional maturity; defined by low levels of intracellular chloride and resting calcium. These neurons are responsive to a number of pharmacological stimuli, but the pharmacological responsiveness of these neurons changes as they mature: early in the course of maturation individual neurons are likely to respond to any of the neurotransmitters, ATP, glutamate, noradrenaline, and acetylcholine with elevations of intracellular calcium, but as these neurons mature they are likely to become responsive to all four ligands. At maturity the *PITX3^eGFP/w^* neurons show a transcript profile indicative of mDA neurons (including PITX3, tyrosine hydroxylase, and TUJ1). We assessed the ability of a neurotoxin (MPP^+^) as well as neuroinflammatory mediators TNF and prostaglandin E_2_ to promote neuron death and neurite retraction of these cultures. MPP^+^ is a powerful toxin in these cells and TNF, but not PGE2, causes some death as well as neurite retraction. In summary, we show that *PITX3^eGFP/w^* neurons are ideal tools for the investigation of mDA neuron development, the pharmacological characterization of receptor populations, as well as *in vitro* neurodegeneration studies.

## Materials and Methods

### hPSC Culture and PITX3^eGFP/w^ Cell Line Generation Using Zinc-Finger Nucleases

The initial hPSC line (H9) was cultured on MEFs (mitomycin C treated mouse embryonic fibroblasts, 4 × 10^5^ cells cm^-2^) in hPSC media: Dulbecco’s Modified Eagle Medium: Nutrient Mixture F-12 (DMEM/F12, 1:1; Invitrogen, Australia), 20% Knock-Out Serum (Invitrogen, Australia), 1% NEAA, 2 mM GlutaMAX^TM^-I, penicillin 25 U mL^-1^, streptomycin 25 μg mL^-1^, 0.1 mM β-mercaptoethanol and 6 ng mL^-1^ FGF-2. Media was changed daily. hPSC cultures were manually groomed to remove differentiated colonies. Fragments (∼0.5 mm^2^ diameter) were manually passaged every 5–7 days onto freshly prepared MEFs.

To create the *PITX3* homologous recombinant cell line, pluripotent H9 human embryonic stem cells (hESCs) were harvested using Accutase and plated on 0.1% v/v gelatin coated dishes in hPSC media containing Rho-associated protein kinase inhibitor (10 μM, Y-27632; Cellagen Technology, USA). Following 30 min incubation the non-adherent hPSCs were collected, centrifuged (200 ×*g*, 5 min), resuspended (7.5 × 10^6^ cells) in 800 μL of 0.22 μm filtered ice cold PBS and transferred to a 0.4 cm electroporation cuvette (Bio-Rad, Australia) together with 10 μg of custom designed ZFN pairs (Sigma-Aldrich, Australia) and 40 μg of a previously described EGFP targeting vector (Hockemeyer et al., 2009) obtained from Addgene (USA). Electroporation was conducted at 250 V, 500 μF (GenePulser XCell instrument; Bio-Rad, Australia) and the electroporated cells plated on MEFs in hPSC medium containing a ROCK inhibitor for the first 24 h. Individual colonies were incubated with puromycin (0.5 μg mL^-1^, Invitrogen, Australia) for 10–14 days after electroporation, at which time puromycin resistant colonies were manually picked and expanded for screening.

For screening correctly targeted colonies, genomic DNA was prepared as previously described (Nefzger et al., 2012). Pooled colonies were initially screened by PCR using the Expand Long Template PCR System (Roche, Australia). PCR positive clones were then further screened by Southern blotting. Digested genomic DNA was electrophoresed, transferred to Hybond-N^+^ membranes (GE Healthcare, Australia) and hybridized with external probes complementary to sequences upstream of the 5′ homology arm of the vector and downstream of the 3′ homology arm, as well as an internal puromycin probe. Probes were labeled with α-32P-dATP (Perkin Elmer, Australia) using a DECAprimeTM II kit (Ambion, Texas) and hybridized in ULTRAhybTM hybridization buffer (Ambion, Texas). The signal was detected using Kodak BioMax MS film in a BioMax Cassette with BioMax TranScreen HE (Sigma-Aldrich, Australia).

### PITX3^eGFP/w^ Neuron Differentiation and Visualization

For differentiation, *PITX3^eGFP/w^* hPSC fragments were transferred to Geltrex (Stem Cell Technologies, Australia) coated six well-plates in mTeSR-1 (Stem Cell Technologies, Australia) media. After 3 days, or when wells were 50–60% confluent, cells were washed thrice in 1X PBS and grown thereafter in a 1:1 mixture of modified Neurobasal and modified DMEM/F12 medium (N2B27 media; Brewer et al., 1993). Additives to this medium were based on the conditions found in Kriks et al. (2011) and included LDN 193189 (LDN, 100 nM; Axon Medchem, Netherlands) from days 0 to 10, SB 431542 (SB, 10 μM; Sigma-Aldrich, Australia) from days 0 to 5, recombinant mouse Sonic Hedgehog C25II (SHH, 100 ng mL^-1^; R&D Systems, USA), recombinant mouse fibroblast growth factor-8b carrier-free (FGF8, 100 ng mL^-1^; R&D Systems, USA), and purmorphamine (PUR, 2 μM; Santa Cruz Biotechnology, USA) from days 1 to 7, CHIR 99021 (CHIR, 3 μM; Tocris Bioscience, USA) from days 3 to 13, and recombinant human glial cell line-derived neurotrophic factor (GDNF (20 ng mL^-1^; R&D Systems, USA), recombinant human BDNF (20 ng mL^-1^; R&D Systems, USA), ascorbic acid (AA, 200 nM; Sigma-Aldrich, USA), DAPT (10 nM; Sigma-Aldrich, USA), N^6^, 2′-*O*-dibutyryladenosine-3′, 5′-cyclic monophosphate (db-cAMP, 0.5 mM; Biolog Life Science Institute, Germany), and recombinant human TGF-β 3 (TGF-β, 1 ng mL^-1^; R&D Systems, USA) from day 10 onward. Cells could be grown on the Geltrex plates until at least day 80 of differentiation (with every-other-day media changes); alternatively after day 20 fragments of cells (roughly 2–3 mm in diameter) could be gently cut away from growing colonies using the tip of a P200 pipette and transferred to laminin (1 mg mL^-1^) coated plates. Colonies were grown in supplemented N2B27 (as described above) until experimentation. eGFP fluorescence during differentiation was confirmed using flow cytometry (below), and visualized with a Nikon A1R confocal microscope (Nikon Instruments, USA).

### Flow Cytometry and Sorting

To characterize differentiating cultures, starting at day 0 of differentiation and continuing at 3 days intervals until day 21, cells were dissociated using Accutase (Sigma-Aldrich, Australia), and resuspended in 1X HBSS (Invitrogen, Australia) containing 10% FCS, and 2 mM glucose at 37^∘^C for cytometric analysis of eGFP fluorescence. Cell suspensions were strained (40 μm; BD Biosciences, Australia) to minimize clumps, and incubated with 5 nM SYTOX Blue (Invitrogen, Australia) to stain for dead cells. Analysis was performed on a FACS Canto II flow cytometer (BD Biosciences, Australia). eGFP fluorescence exclusion gating was employed to ensure that >99% of the wild type control population (also subjected to the same staining) were eGFP-negative. The key to neuron survival using this approach is to have as low a pressure, and as wide a nozzle as possible.

Cells were also FACS sorted for mRNA extraction and subsequent qPCR. On days 0, 20, 40, 60, and 80, cells were dissociated using accutase, strained, and resuspended as above. FACS was performed using a MoFlo Astrios sorter (Beckman Coulter Inc., USA) and cells divided into eGFP positive and eGFP negative fractions. The positive and negative fractions from the aggregates of three independently differentiating wells (to ensure sufficient cells for each sort) were centrifuged at 200 ×*g* for 5 min and the supernatant removed prior to RNA extraction.

### RNA Extraction and Quantitative-PCR

Total RNA was extracted from 10^6^ cells using the RNEasy Minikit according to manufacturer’s instructions (Qiagen, Australia). Samples were analyzed for RNA content using the Nanodrop ND-1000 (Thermo Scientific, USA) spectrophotometer. Three technical replicate qPCR reactions were performed on samples aggregated from three independently differentiating wells using the iScript One-Step RT-PCR Kit with SYBR-Green according to the manufacturer’s specifications (Bio-Rad, Australia). qPCR reactions and primer sequences are described in **Table 1**. Relative quantification of gene expression during differentiation was obtained using the 2^-ΔΔCt^ method (Livak and Schmittgen, 2001) by comparing Ct values of target genes to mean Ct values of two housekeeping genes, β-actin and GAPDH.

**Table 1:** Quantitative polymerase chain reactions (qPCRs) were performed in the C1000 Thermal Cycler coupled to a CFX96 Real Time System (Bio-Rad, Australia).

Gene	Fwd/Rev	Sequence 5′ to 3′	Melt ^∘^C
*TH*	Fwd	CGG GCT TCT CGG ACC AGG TGT A	73.2
	Rev	CTC CTC GGC GGT GTA CTC CAC A	72.6
*AADC*	Fwd	CTC GGA CCA AAG TGA TCC AT	63.9
	Rev	GTC TCT CTC CAG GGC TTC CT	63.8
*NURR1*	Fwd	TTC TCC TTT AAG CAA TCG CCC	66.1
	Rev	AAG CCT TTG CAG CCC TCA CAG	69.8
*PITX3*	Fwd	GCC AAC CTT AGT CCG TG	59.2
	Rev	GCA AGC CAG TCA AAA TG	57.8
*DAT*	Fwd	AGC AGA ACG GAG TGC AGC T	65.4
	Rev	GTA TGC TCT GAT GCC GTC T	60.5
*FOXG1*	Fwd	TGG CCC ATG TCG CCC TTC CT	75.1
	Rev	GCC GAC GTG GTG CCG TTG TA	73.5
*NKX2.1*	Fwd	AGG GCG GGG CAC AGA TTG GA	75.1
	Rev	GCT GGC AGA GTG TGC CCA GA	71.4
*PAX5*	Fwd	CCC CAT TGT GAC AGG CCG TGA C	74.8
	Rev	TCA GCG TCG GTG CTG AGT AGC T	70.5
*EN1*	Fwd	TCT CGC TGT CTC TCC CTC TC	63.9
	Rev	CGT GGC TTA CTC CCC ATT TA	63.6
*WNT1*	Fwd	GAG CCA CGA GTT TGG ATG TT	64.0
	Rev	TGC AGG GAG AAA GGA GAG AA	64.0
*GIRK2*	Fwd	GCT ACC GGG TCA TCA CAG AT	63.9
	Rev	ACT GCA TGG GTG GAA AAG AC	63.9
*LMX1A*	Fwd	CGC ATC GTT TCT TCT CCT CT	63.4
	Rev	CAG ACA GAC TTG GGG CTC AC	65.0
*FOXA2*	Fwd	GGG GTA GTG CAT CAC CTG TT	63.8
	Rev	CCG TTC TCC ATC AAC AAC CT	63.9
*SHH*	Fwd	CCA ATT ACA ACC CCG ACA TC	63.9
	Rev	AGT TTC ACT CCT GGC CAC TG	64.3
*CORIN*	Fwd	CAT ATC TCC ATC GCC TCA GTT G	65.6
	Rev	GGC AGG AGT CCA TGA CTG T	62.9
*HOXA2*	Fwd	CGT CGC TCG CTG AGT GCC TG	74.1
	Rev	TGT CGA GTG TGA AAG CGT CGA GG	72.7
*ACTB*	Fwd	CCT TGC ACA TGC CGG AG	66.9
	Rev	GCA CAG AGC CTC GCC TT	64.6
*GAPDH*	Fwd	TTG AGG TCA ATG AAG GGG TC	63.9
	Rev	GAA GGT GAA GGT CGG AGT CA	64.6
*OCT4*	Fwd	TCT CCA GGT TGC CTC TCA CT	64.1
	Rev	GTG GAG GAA GCT GAC AAC AA	62.9
*MAP2*	Fwd	CCG TGT GGA CCA TGG GGC TG	75.3
	Rev	GTC GTC GGG GTG ATG CCA CG	75.4
*SYP*	Fwd	ACC TCG GGA CTC AAC ACC TCG G	72.6
	Rev	GAA CCA CAG GTT GCC GAC CCA G	73.8

*Reaction conditions included cDNA synthesis (10 min at 50^∘^C), reverse transcriptase inactivation (5 min at 95^∘^C), PCR cycling (10 s at 95^∘^C then 30 s at 55–60^∘^C; up to 43 cycles), and melt curve analysis (1 min at 95^∘^C, 1 min at 55^∘^C, and 10 s each at steps of 0.5^∘^C between and 55–95^∘^C). Primers used in gene expression assay.*

### Immunocytochemistry

Immunocytochemistry was carried out as previously described (Khaira et al., 2009). Briefly, cells were washed free of PBS/sodium azide (3 × 5 min in PBS) and permeabilized with 0.1% Triton X-100 (Sigma-Aldrich, Australia) in PBS for 30 min at room temperature. Cells were then blocked with 1% normal donkey serum in PBS for 30 min and incubated overnight in 0.1% Triton X-100 in PBS with the primary antibodies, anti-tyrosine hydroxylase (TH; rabbit IgG, 1:200; Millipore, Australia), anti-βIII tubulin (mouse IgG, 1:1000; Covance, Australia), anti-FOXA2 (mouse IgG, 1:1000; DHSB, USA), anti-GABA (rabbit IgG, 1:200; Sigma-Aldrich, Australia), anti-GIRK2 (rabbit IgG, 1:200; Alomone labs, Israel), and anti-LMX1A (rabbit IgG, 1:200; Sigma-Aldrich, Australia). Cells were then incubated with the secondary antibodies donkey anti-mouse Alexa Fluor 488 and donkey anti-rabbit Alexa Fluor 594 (both Molecular Probes, USA) at 1:1000 for 2 h at room temperature. Cells were visualized using a Nikon TE2000U microscope coupled to a SPOT RT camera (for post live-cell imaging immunocytochemistry) or a Nikon A1R confocal microscope (for other immunocytochemistry; Nikon, Japan). Fields of view corresponding with those used for Ca^2+^ and Cl^-^ ion imaging studies were identified by aligning initial images with those taken after immunolabeling. TH immunoreactive and eGFP positive (TH^+^eGFP^+^) neurons were identified and regions defined so that [Ca^2+^]_i_ and [Cl^-^]_i_ responses in those neurons could be determined after replaying the initial live cell experiments.

### Live Cell Calcium and Chloride Ion Imaging

Calcium and chloride ion imaging was performed on days 20, 40, 60, and 80 as described previously (Haynes et al., 2002; Watmuff et al., 2012). For calcium imaging; neurons were incubated in HEPES buffered salt solution (consisting of NaCl, 145 mM; KCl, 5 mM; MgSO_4_, 1 mM; HEPES, 10 mM; CaCl_2_, 2 mM; glucose, 10 mM, containing 0.1% bovine serum albumin, at pH 7.4) in the presence of 5 μM FURA-2AM (ICN Biochemicals) for 40 min. Cells were then allowed to hydrolyze the acetoxymethyl ester for 40–60 min (37^∘^) prior to viewing with a Nikon TE2000U microscope coupled to a SPOT RT camera (for post live-cell imaging immunocytochemistry) or a Nikon A1R confocal microscope. Cell temperature was maintained at near 37^∘^C with a heated stage. A DG4 (Sutter, USA) was used to illuminate cells with light at 340 and 380 nm. Cell fluorescent emission at 510 nm was recorded every 1–2 s. Background emission was subtracted from each image, and 340/380 ratios of the resultant intensity emission values at each time point were obtained.

For intracellular calcium quantitation, the calcium concentration was calculated using the equation (Grynkiewicz et al., 1985):

$${\left[{Ca}^{2+}\right]}_{i}=K_{D}\beta \left[\left(R-R_{\min}\right)/\left(R_{\max}-R\right)\right]$$

Where β is the emission ratio of R_min_/R_max_ at 380 nm. The dissociation constant (*K_D_*) value of 285 nM was taken from (Groden et al., 1991). The R_min_ value was obtained in the absence of Ca^2+^ and in the presence of both 4-Br-A232187 (20 μM) and EGTA (10 mM). The R_max_ value was obtained in the presence of both 4-Br-A232187 (20 μM) and Ca^2+^ (10 mM).

For non-quantitative calcium imaging neurons were loaded with Fluo4AM (10 μM 30 min) as described previously (Khaira et al., 2011). Briefly, following a 30 min equilibration period minute equilibration period and 10 min of baseline imaging either of the agonists adenosine triphosphate (ATP, 300 μM), noradrenaline (NA, 30 μM), acetylcholine (ACh, 30 μM), L-glutamate (Glut, 30 μM), γ-aminobutyric acid (GABA, 30 μM; all Sigma-Aldrich, Australia), We have previously shown these concentrations to be effective in elevating [Ca^2+^]_i_ in mDA neurons (Lang et al., 2004; Raye et al., 2007; Khaira et al., 2011) Following peak response or after 5 min, wells were washed three times with PBS and preparations allowed at least 5 min to recover before the addition of the next agonist. This was repeated until all five agonists had been added. After the final agonist addition wells were washed and KCl (30 mM) added.

For voltage operated calcium channel inhibitor studies cells were loaded with Fluo-4AM (as described above). Following a 10 min equilibration period KCl (30 mM) was added to culture plates and changes in fluorescence intensity recorded over 30 s. Preparations were then washed and nifedipine (10 μM) was added, 5 min later KCl was added again and changes in fluorescence intensity recorded. Preparations were washed and nifedipine re-added, along with ω-conotoxin (0.4 μM) and responses to KCl measured again. This process was repeated with the addition of mibefradil (10 μM). Control responses to KCl (30 mM, x4) were measured in the presence voltage operated calcium channel inhibitor vehicle.

For chloride ion imaging we used the method previously described (Watmuff et al., 2012). Briefly, we incubated neurons with the Chloride-sensitive fluorophore dihydro-MEQ (10 μM; Inglefield and Schwartz-Bloom, 1999) in normal HEPES buffer, measuring fluorescence intensity following excitation at 340/26 nm; emission was recorded at 510/84 nm. Resting state fluorescence and fluorescence following the addition of GABA (30 μM) were recorded. We changed to a Cl^-^ free buffer (normal HEPES with gluconate ions replacing Cl^-^) containing nigericin (10 μM), tributyltin (10 μM), and valinomycin (5 μM) to measure minimum fluorescence. 10 min later Cl^-^ was added back into the tissue buffer at 0.1, 1, 10, and 100 mM, allowing a Stern–Volmer relationship to be constructed (where F_0_/F denotes fluorescence in the absence of chloride divided by fluorescence in the presence of chloride), this relationship was used to construct a chloride ion standard curve from which neuron [Cl^-^]_i_ was calculated (Krapf et al., 1988).

Following each experiment cells were fixed 4% (w/v) paraformaldehyde in PBS for 25 min at room temperature and then stored in PBS with 0.1% sodium azide (Sigma-Aldrich, Australia) until used for immunocytochemistry.

### Dopamine ELISA

On days 20, 40, 60, and 80 of differentiation neurons were incubated with HEPES buffer (37^∘^C) containing nipecotic acid (6 μM) and AA (200 μM). The HEPES buffer was collected from each well 60 s after the application of KCl (30 mM), GABA (30 μM), Glut (30 μM), or vehicle. The order of addition of agents was randomized in each experiment. Following collection, samples were immediately frozen by placing vials on dry ice and then stored in liquid nitrogen until use. ELISA was performed using the dopamine ELISA kit (Genway Biotech., USA) according to the manufacturer’s instructions. Optical density at 405 nm was measured immediately after the reaction was stopped using an EnVision 2.101 multilabel fluorescence plate reader (PerkinElmer, Australia) and dopamine concentrations of samples were interpolated using the standard curve generated.

### Neurodegeneration Assay

Cells were differentiated for 80–90 days. eGFP was visualized using a Nikon A1R confocal microscope equipped with an environmental chamber to allow neuron survival. Z-plane stacks of *PITX3^eGFP/w^* neurons were taken every 2 h for up to 48 h. Ligands were added immediately following the first round of imaging (time = 0 h). Cell loss was defined by the fraction of eGFP fluorescence remaining in each field of view following the addition of MPP^+^ (50 μM), TNF (20 ng mL^-1^), PGE_2_ (300 nM), or vehicles. In some experiments the cell impermeant nuclear acid stain TO-PRO^®^-3 (1 nM; Invitrogen, Australia) was added to wells to establish when neuron membranes became permeable. *PITX3^eGFP/w^* neuron-containing fields of view were analyzed using Nikon Elements imaging software (Nikon, Japan). For analysis of neuron death, stacks of images were used to create maximum intensity projection images, which were then thresholded for eGFP. Given the presence of fluorescent beads developing in cultures images, especially after the addition of TNF, small fluorescent objects (with areas of less than <50 μm^2^) were excluded from analysis prior. The total fluorescence area was then calculated before and after ligand and used to generate a ratio (post-ligand/pre-ligand). We also measured neurite length over time using the maximum intensity projection images, single cells with clear eGFP^+^ projections were identified from pre-ligand images and the neurites manually measured with a tracing tool. For this study we found the 2 h imaging to be invaluable since it enabled us to trace neurite outgrowth/retraction, even as the soma moved around the plate.

### Statistical Analysis

Results are presented as the arithmetic mean ± standard error of the mean (SEM) of at least three independent experiments. Statistical analyses were performed using GraphPad Prism v5.00 and later v6.00 (GraphPad Software, USA) and employed a Student’s *t*-test (two-tailed), or one-way analysis of variance (ANOVA) with *post hoc* Bonferroni’s or Dunnett’s test as appropriate. Paired *t*-tests were used when treatments allowed for ‘before and after’ measurements of cell responses in live cell experiments. For experiments involving single cell measures of fluorescence intensity, *n* = 3, 60 cells indicates that changes were measured in 60 cells spread across three (temporally distinct) independent differentiations (∼20 cells per differentiation).

## Results

### Generation of hPSC Reporter Line and Differentiation of PITX3^eGFP/w^ Neurons

**FIGURE 1:**
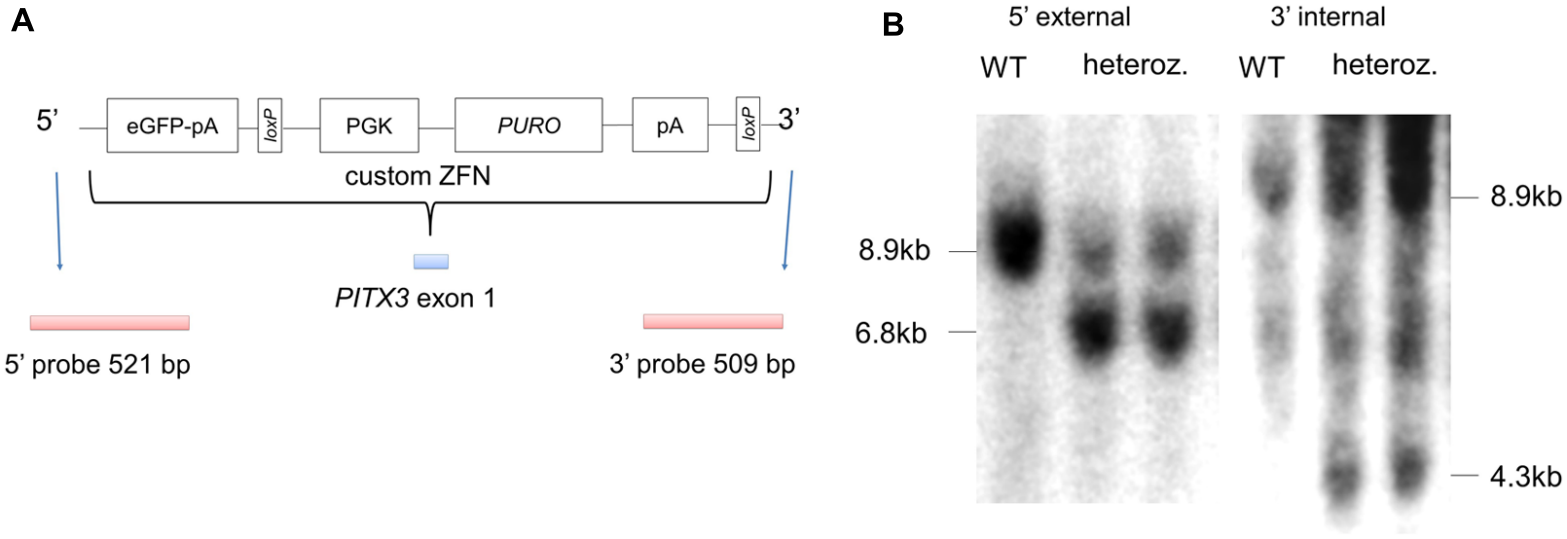
**Targeting eGFP to the endogenous PITX3 locus in hPSCs using zinc finger nucleases (ZFN).** **(A)** shows a schematic of the donor plasmid for targeting eGFP to the endogenous PITX3 locus; the strategy employed was based on that reported by Hockemeyer et al. (2009). PGK, human phosphoglycerol kinase promoter; PURO, puromycin resistance gene; loxP, loxP sites; pA, polyadenylation sequence. **(B)** Correctly targeted colonies (as determined by PCR) were generated with 19% efficiency, and subsequently confirmed by Southern blot analysis (modified from Hartley et al., 2014).

**FIGURE 2:**
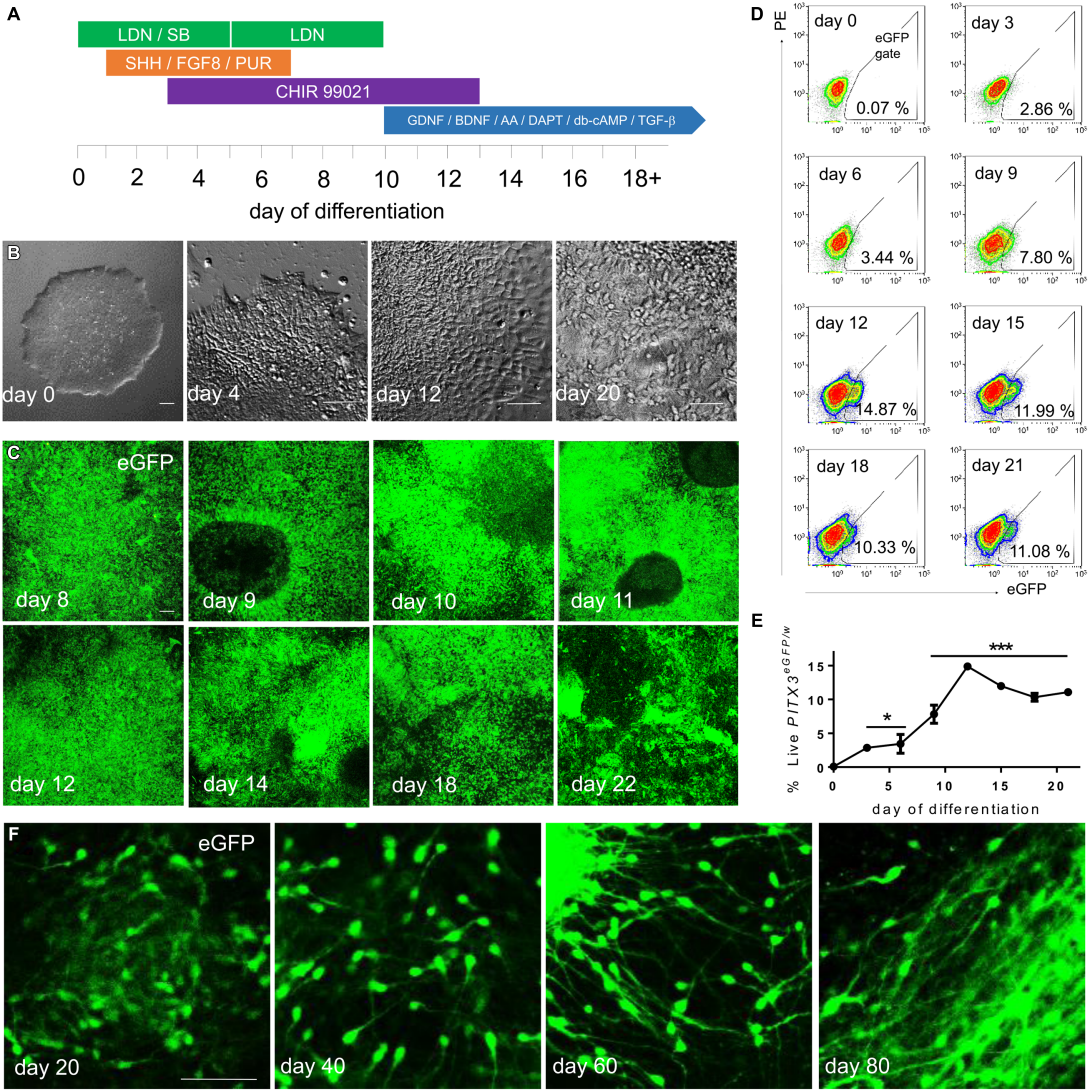
***PITX3^**eGFP/w**^*cells appear during chemically defined monolayer differentiation**. **(A)** *PITX3^eGFP/w^* hPSCs were transferred in fragments to Geltrex coated plates in mTeSR media. After 3 days, or when wells became 50–60% confluent, differentiation was initiated in N2B27 media using containing LDN 193189 (LDN), SB 431542 (SB), recombinant mouse/human Sonic Hedgehog C25II (SHH), recombinant mouse fibroblast growth factor-8b (FGF8), purmorphamine (PUR), CHIR 99021 (CHIR), recombinant human glial cell line-derived neurotrophic factor (GDNF), recombinant human brain cell line-derived neurotrophic factor (BDNF), ascorbic acid (AA), DAPT, N^6^, 2′-*O*-dibutyryladenosine-3′, 5′-cyclic monophosphate (db-cAMP), and recombinant human TGF-β 3 (TGF-β). **(B)** Cell morphology transitioned from PSC fragments to immature neurons by day 20. **(C)** Cell bodies containing eGFP could be seen developing across cultures from day 8 to day 22 of differentiation. **(D)** The percentages of live eGFP^+^ cells in culture were observed using flow cytometry. **(E)** shows the percentage of live eGFP^+^ cells to day 21 (one-way ANOVA with *post hoc* Dunnett’s test compared to day 0, ^∗^ indicates *p* < 0.05 and ^∗∗∗^ indicates *p* < 0.001, *n* = 3). **(F)** Upon extended differentiation, eGFP^+^ cells with neuronal morphology were seen in culture until at least day 80. Scale bars; 100 μm.

A ZFN pair together with a targeting vector was used to introduce an eGFP-puromycin cassette to pluripotent H9 hESCs at exon 1 of the endogenous PITX3 locus (**Figure 1A**). Targeting efficiency was 19% of total colonies picked and was confirmed with southern blot analysis (**Figure 1B**). A modified version of a recently described floor plate differentiation protocol (Kriks et al., 2011; summarized in **Figure 2A**) was used to derive mDA neurons. Neural differentiation was evident by the polarization of cell bodies and increased size of initial colonies by day 4 (**Figure 2B**). By day 12 of differentiation wells were largely confluent, by day 20, small (10–20 μm) polar neuronal cell bodies could be seen (**Figure 2B**). From day 8 eGFP fluorescence positive cells were visible (**Figure 2C**). Wild type H9 cells differentiated under the same conditions did not show any eGFP fluorescence, nor did *PITX3^eGFP/w^* hPSCs differentiated without fate directing morphogens (i.e., SHH, FGF8, or CHIR; data not shown). Flow cytometry was used to quantify the emergence of eGFP during differentiation (**Figure 2D**). The number of eGFP^+^ cells present per well-increased from 0 at day 0 to 14.9 ± 0.3% by day 12, this remained stable until day 21 (**Figure 2E**; one-way ANOVA with *post hoc* Dunnett’s test compared to day 0, *p* < 0.05 and 0.001, *n* = 3).

### Differentiated PITX3^eGFP/w^ Cells Display Transcript, Protein, and Functional Capabilities of Midbrain Dopaminergic Neurons

**FIGURE 3:**
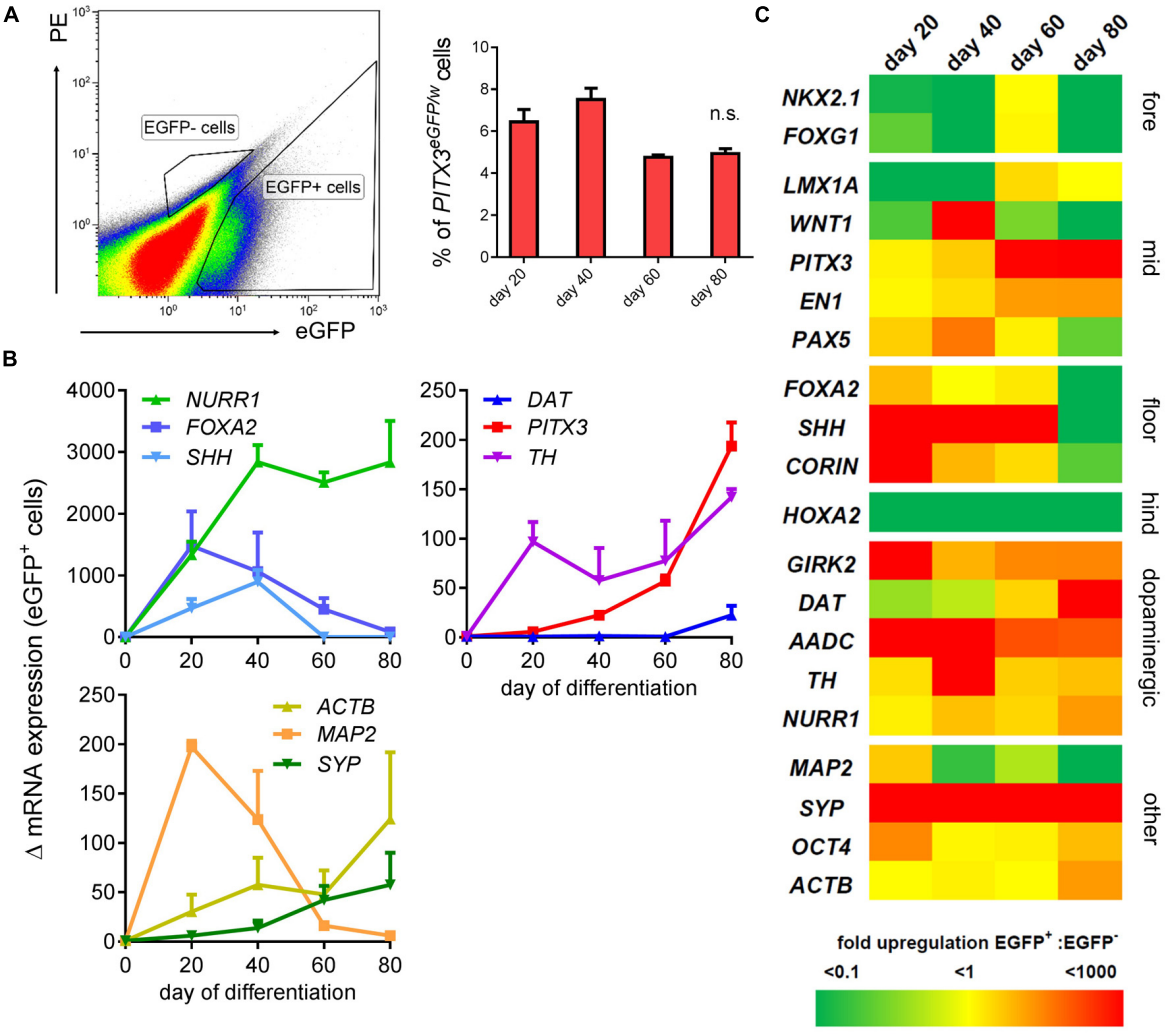
**Transcriptional identity of* PITX3^**eGFP/w**^* cells is midbrain dopaminergic.** A second flow cytometry experiment was conducted to collect eGFP^+^ and eGFP^-^ cells for total RNA extraction and qPCR analysis on days 20, 40, 60, and 80. **(A)** Shows a typical FACS plot at day 20, and the strategy used to isolate eGFP^+^ and eGFP^-^ cells. This graph shows the percentage of live cells (from three independent experiments; ± SEM) sorted into the eGFP^+^ gate at each time point. **(B)** qPCR analysis of the mRNA from at least 10^6^ eGFP^+^ cells showed upregulation of neuronal and midbrain dopaminergic genes (compared to hPSCs; one-way ANOVA with *post hoc* Dunnett’s test, *p* < 0.001-0.05, *n* = 3 technical replicates of samples aggregated from three independently differentiating wells) at points between days 20 and 80 of differentiation. The *y*-axis shows the fold change in mRNA levels (Δ mRNA).** (C)** Shows a heat map of the ratio of mean transcript levels in the positive fraction : negative fraction. Fore, forebrain; mid, midbrain; floor, floorplate; hind, hindbrain.

Maturation of cultures by re-plating onto laminin coated wells revealed bright eGFP^+^ cell bodies clearly evident from days 20 to 80 of differentiation (**Figure 2F**). These cells displayed neuronal morphology and possessed eGFP throughout their soma and associated processes (**Figure 2F**). Comparative gene expression analysis via quantitative PCR (qPCR) was performed on eGFP positive and negative fractions (plot shown in **Figure 3A**) collected via FACS at day 20, 40, 60, and 80 of differentiation. Between 4 and 8% live eGFP^+^ cells were collected on each sort (**Figure 3A**). eGFP^+^ sorted cells revealed an up-regulation of markers of the dopaminergic neuron phenotype; *DAT*, *AADC*, and *TH* as well as the floor plate markers *FOXA2*, *SHH*, *CORIN*, and the midbrain markers *LMX1A*, *WNT1*, *EN1*, *PITX3,* and *PAX6* at all time points compared to hPSCs (**Figure 3B**; one-way ANOVA with *post hoc* Dunnett’s test, *p* < 0.05, *n* = 3). eGFP^+^ cells also showed high levels of the neuronal marker *MAP2*, and the synaptic marker *SYP* (**Figure 3B**; one-way ANOVA, *p* < 0.05, *n* = 3). The forebrain markers *NKX2.1* and *FOXG1*, as well as the hindbrain marker *HOXA2*, were predominantly expressed in the eGFP^-^ fraction (**Figure 3C**). *SYP* and *MAP2* enrichment were also found in the eGFP^-^ fraction (**Figure 3C**).

**FIGURE 4:**
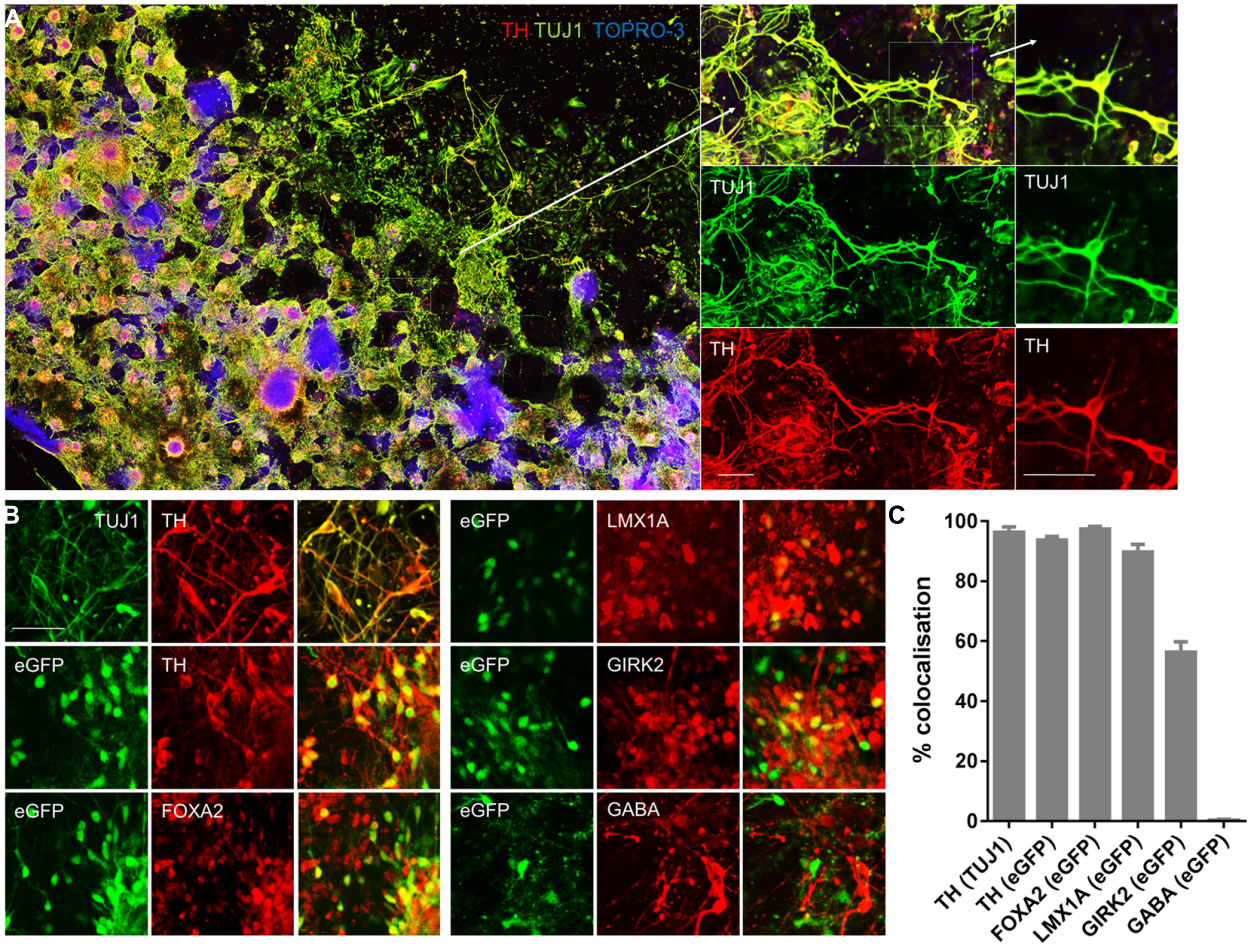
***PITX3^eGFP/w^* cells express protein markers of midbrain dopaminergic neurons.** Immunocytochemical analysis of *PITX3^eGFP/w^* cultures was performed at day 20 in order to confirm the presence of an mDA neuronal phenotype. **(A)** Wells were replete with the neuronal marker β-III tubulin (TUJ1; green), which was almost always co-localized with the dopaminergic marker tyrosine hydroxylase (TH; red). The panels, from left to right, show low and high powered color-merges of TUJ1 and TH, in combination with the nuclear stain TO-PRO-3 (blue). **(B)** Immunocytochemical analysis of *PITX3^eGFP/w^* cultures was performed at day 20 in order to confirm the presence of an mDA neuronal phenotype. Populations of *PITX3^eGFP/w^* cells expressed mDA markers TH, FOXA2, LMX1A, and the A9 group marker GIRK2, but not the GABAergic marker GABA. **(C)** Shows the relative co-localization of protein markers at day 20. The columns represent the percentage of cells positive for both markers (data presented as mean ± SEM, *n* = 3 fields of view from independent wells). Scale bars; 100 μm.

Next, we confirmed transcriptional profiling of eGFP^+^ cells using immunocytochemistry. Confocal microscopy at day 20 revealed abundant expression of the neuronal cytoskeletal protein β-III tubulin (TUJ1) as well as TH (**Figure 4A**). 96 ± 2% of TH^+^ cells were TUJ1^+^ (**Figures 4B,C**). eGFP^+^ cells also co-expressed TH (94 ± 2% of eGFP^+^ cells), FOXA2 (97 ± 1% of eGFP^+^ cells), LMX1A (90 ± 3% of eGFP^+^ cells), and the K^+^ inward-rectifying channel, GIRK2 (56 ± 4% of eGFP^+^ cells; (**Figures 4B,C**). GABA immunoreactivity was widespread from day 20 onward, but was rarely co-localized with eGFP (1 ± 1%; **Figures 4B,C**).

### Functional Properties of PITX3^eGFP/w^ Neurons Develop During *In Vitro* Differentiation

**FIGURE 5:**
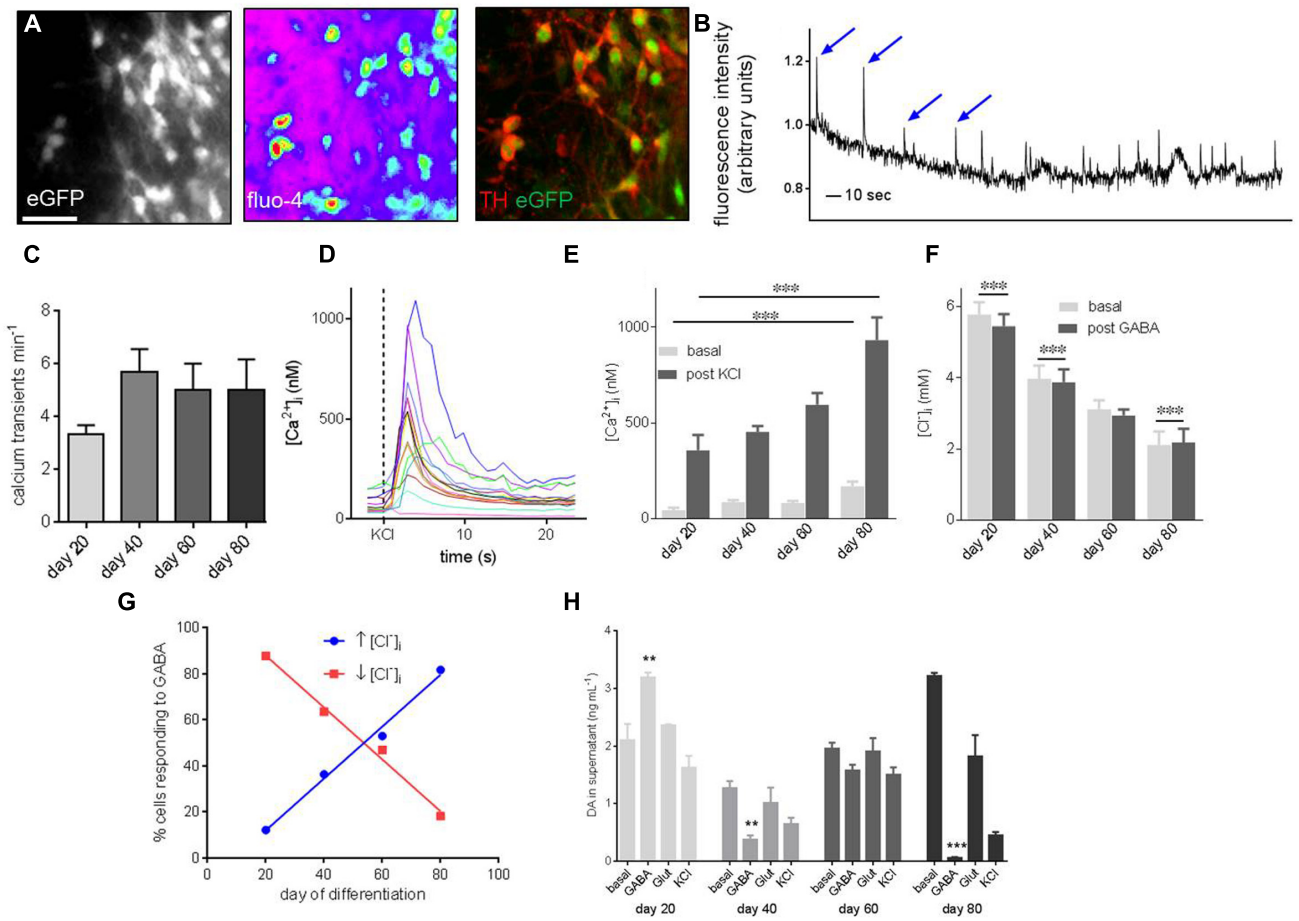
**Functional characterization of* PITX3^**eGFP/w**^* neurons reveals development of maturity during terminal differentiation.** **(A)** Ca^2+^ and Cl^-^ imaging was employed to probe the function of *PITX3^eGFP/w^* neurons. These panels show, from left to right, eGFP in a field of view prior to imaging, the Ca^2+^ -sensitive probe Fluo-4 loaded into cells of the same field, and TH and eGFP after post-imaging immunocytochemistry. **(B)** Shows a representative trace from a Ca^2+^ imaging experiment. Spontaneous oscillations of [Ca^2+^]_i_, typical of *in vitro* mDA neurons are highlighted by arrows. The *y*-axis represents change in fluorescence: baseline fluorescence (ΔF/F). **(C)** The frequency of these oscillations did not change significantly by day 80 (one-way ANOVA with *post hoc* Dunnett’s test, indicates *p* > 0.05, *n* = 3 wells, 60–80 cells). **(D)** Intracellular calcium ion concentration ([Ca^2+^]_i_) in *PITX3^eGFP/w^* neurons was calculated at rest and after stimulation with KCl (30 mM). **(E)** Basal and KCl-induced elevations of [Ca^2+^]_i_ increased during differentiation (one-way ANOVA with *post hoc* Dunnett’s test, ^∗∗∗^ indicates *p* < 0.001, *n* = 3 experiments, 40–80 cells). **(F)** Similarly, GABA (30 mM) decreased [Cl^-^]_i_ at day 20 and 40 of differentiation, by day 80 GABA induced a significant increase in [Cl]_i_ (Student’s paired *t*-test, ^∗∗∗^ indicates *p* < 0.001, *n* = 4, 60–80 cells). **(G)** By day 80 most *PITX3^eGFP/w^* neurons respond to GABA with an elevation of [Cl^-^]_i_ (blue; *R*^2^ = 0.99), rather than the decrease seen at earlier days of differentiation (red; *R*^2^ = 0.99). **(H)** Shows the change in dopamine concentration in the supernatant of eGFP^+^ cultures following vehicle addition (i.e., basal level), GABA (30 mM), l-glutamate (30 mM) or KCl (30 mM) at different days of culture. Dopamine release in response to GABA (30 μM), but not l-glutamate or KCl significantly decreased from days 20 to 80 of differentiation (one-way ANOVA with *post hoc* Dunnett’s test compared to day 20, ^∗∗^, ^∗∗∗^ indicates *p* < 0.01 and 0.001, respectively, *n* = 3 wells).

To determine whether *PITX3^eGFP/w^* neurons were functional we performed live-cell fluorescent imaging to measure intracellular calcium ([Ca^2+^]_i_) and chloride ion ([Cl^-^]_i_) activity, as well as DA release during differentiation (**Figure 5A**). From day 20 around 20% of *PITX3^eGFP/w^* neurons (which were subsequently shown to be immunoreactive to TH) showed regular spontaneous elevations of [Ca^2+^]_i_ (**Figure 5B**) at a frequency of 3.3 ± 0.3 min^-1^ (**Figure 5C**). By day 80 the frequency of these events was 5.0 ± 0.1 min^-1^ (**Figure 5C**; change from days 20 to 80 was not significant, one-way ANOVA with *post hoc* Dunnett’s test, *p*> 0.05, *n* = 3). [Ca^2+^]_i_ in *PITX3^eGFP/w^* neurons was calculated at rest and after stimulation with KCl (30 mM; **Figure 5D**). Basal [Ca^2+^]_i_ increased during differentiation from 45 ± 13 nM at day 20 to 169 ± 25 nM at day 80 (**Figure 5E**; one-way ANOVA, *p* < 0.001, *n* = 4, 60–80 cells). KCl (30 mM) increased maximal [Ca^2+^]_i_ from 357 ± 80 nM at day 20 to 931 ± 119 nM at day 80 (**Figure 5E**; one-way ANOVA, *p* < 0.001, *n* = 4, 60–80 cells).

[Cl^-^]_i_ was calculated in *PITX3^eGFP/w^* neurons at rest and after stimulation with GABA (30 μM; **Figure 5F**). Basal [Cl^-^]_i_ decreased during differentiation from 5.8 ± 0.4 mM at day 20 to 2.1 ± 0.4 mM at day 80 (**Figure 5F**; one-way ANOVA, *p* < 0.001, *n* = 4, 60–80 cells). At day 20, GABA significantly decreased basal [Cl^-^]_i_, but by day 80 GABA elevated basal [Cl^-^]_i_ (Student’s paired *t*-test, *p* < 0.001, *n* = 4, 60–80 cells). The proportion of *PITX3^eGFP/w^* neurons responding to GABA with an elevation of [Cl^-^]_i_ increased from 12% at day 20 to 82% at day 80 (**Figure 5G**).

Cultures containing *PITX3^eGFP/w^* neurons released DA both at rest, and following pharmacological stimulation using GABA (30 μM), Glut (30 μM), and KCl (30 mM; **Figure 5F**). Basal release of DA in culture increased during differentiation from 2.1 ± 0.3 ng mL^-1^ at day 20 to 3.22 ± 0.05 ng mL^-1^ at day 80 (**Figure 5F**; one-way ANOVA with *post hoc* Dunnett’s test, *p* < 0.01, *n* = 3). DA release following stimulation with GABA (30 μM) was 3.93 ± 0.86 ng mL^-1^ at day 20. By day 40, post-GABA DA release decreased and remained constant until day 80 (0.05 ± 0.16 ng mL^-1^; **Figure 5H**; one-way ANOVA with *post hoc* Dunnett’s test, *p* < 0.001, *n* = 3).

**FIGURE 6:**
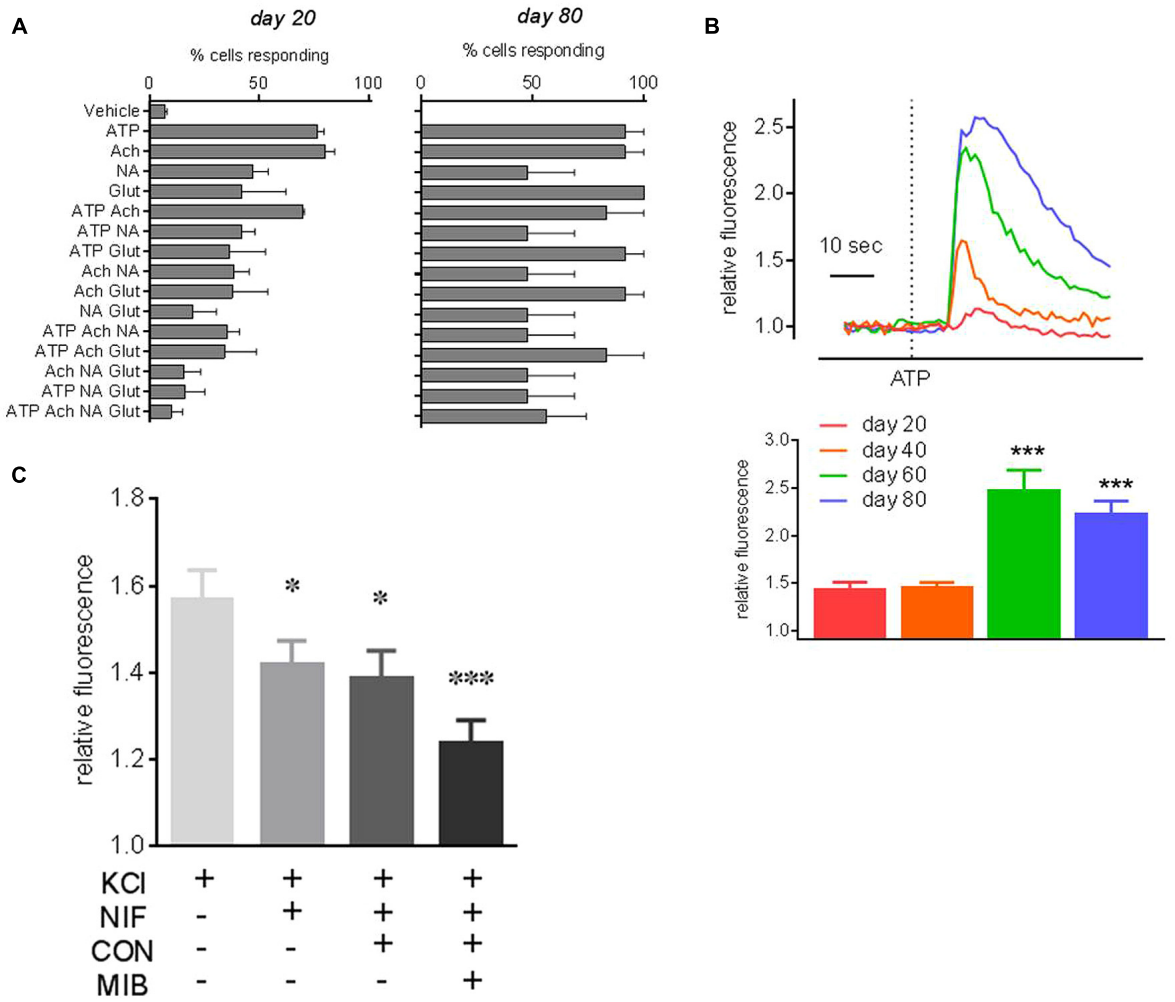
**Neuropharmacology of *PITX3^eGFP/w^* neurons.** Ca^2+^ imaging of eGFP^+^ cells revealed functional neurons responding to neurotransmitters changes over time. **(A)** Shows the percentages of eGFP^+^ cells, at days 20 and 80 of differentiation, that respond to pharmacological stimuli with increases of [Ca^2+^]_i_ >10 SD of baseline (mean ± SEM, *n* = 3). ATP, adenosine triphosphate; NA, noradrenaline; Glut, l-glutamate; ACh, acetylcholine; or V, vehicle. For example, at day 20 very few eGFP^+^ cells respond to all of ATP, NA, Ach and glutamate, this figure increases to ∼50% of neurons at day 80. **(B)** Shows typical *PITX3^eGFP/w^* neuron [Ca^2+^]_i_ responses to ATP on days 20–80. Mean fold changes in [Ca^2+^]_i_-related fluorescence in response to ATP (±SEM) are shown underneath. **(C)** Shows the effect of the cumulative addition of calcium channel inhibitors, nifedipine (10 μM), ω-conotoxin MVIIA (0.4 μM), and mibefradil (10 μM) on the KCl-induced change in [Ca^2+^]_i_ at day 80. KCl (30 mM) control responses in the presence of voltage operated calcium channel inhibitor vehicles did not significantly change throughout the experiment. All data was analyzed using a one way ANOVA with *post hoc* Dunnett’s test compared to vehicle or day 20 data. (^∗^ and ^∗∗∗^ indicates *p* < 0.05 and 0.001, *n* = 3).

Next, the ability of *PITX3^eGFP/w^* neurons to respond to common neurotransmitters was investigated using Fluo-4 Ca^2+^ imaging. At days 20, 40, 60, and 80 *PITX3^eGFP/w^* cells responded to ATP (300 μM), GABA (30 μM), KCl (30 mM), NA (30 μM), Glut (30 μM), ACh (30 μM), and Ca^2+^ (10 mM) with elevations of [Ca^2+^]_i_ (percentages of eGFP^+^ cells responding to each neurotransmitter are shown in **Figure 6A**). **Figure 6B** shows the typical responses of cells responding to ATP from each time point, and also the mean (±SEM) increase in Ca^2+^-related fluorescence after ATP at each time point. *PITX3^eGFP/w^* neuron responsiveness to individual agents was close to 100% by day 80; the exception being NA, which elicited elevations of [Ca^2+^]_i_ in <50% of cells (**Figure 6A**). We next looked at the ability of individual *PITX3^eGFP/w^* neurons to respond to multiple agonists in order to ascertain whether they became functionally restricted to specific pharmacological stimuli during maturation. At day 20 only 10 ± 9% of cells responded to all the neurotransmitters (ATP, ACh, NA, and Glut) with elevations of intracellular calcium. By day 80, this figure had risen to 48 ± 21% of all *PITX3^eGFP/w^* neurons (**Figure 6C**; one-way ANOVA with *post hoc* Dunnett’s test, *p* > 0.05, *n* = 3, 40–60 cells).

Lastly, in order to investigate the contribution of voltage operated Ca^2+^ channels to the KCl-induced elevations of [Ca^2+^]_i_ we examined the KCl-induced elevation of [Ca^2+^]_i_ in the absence and presence of three specific voltage operated Ca^2+^ channel inhibitors, added cumulatively, nifedipine (10 μM; L-type blocker), ω-conotoxin MVIIA [0.4 μM; N (primarily)/P/Q type blocker] and mibefradil (10 μM, T type blocker; **Figure 6C**). Nifedipine reduced the KCl-induced elevation of [Ca^2+^]_i_ by 27% (one-way ANOVA with *post hoc* Dunnett’s test, *p* < 0.05, *n* = 3, 60–80 cells). The subsequent addition of ω-conotoxin to nifedipine had no further effect on KCl-induced elevations of [Ca^2+^]_i_, but the addition of mibefradil to the combination of ω-conotoxin and nifedipine further reduced the KCl-induced elevation of [Ca^2+^]_i_ by 59% (**Figure 6C**; one-way ANOVA with *post hoc* Dunnett’s test, *p* < 0.001, *n* = 3, 60–80 cells).

### PITX3^eGFP/w^ Neuron Responses to MPP^+^ and Neuroinflammatory Mediators

**FIGURE 7:**
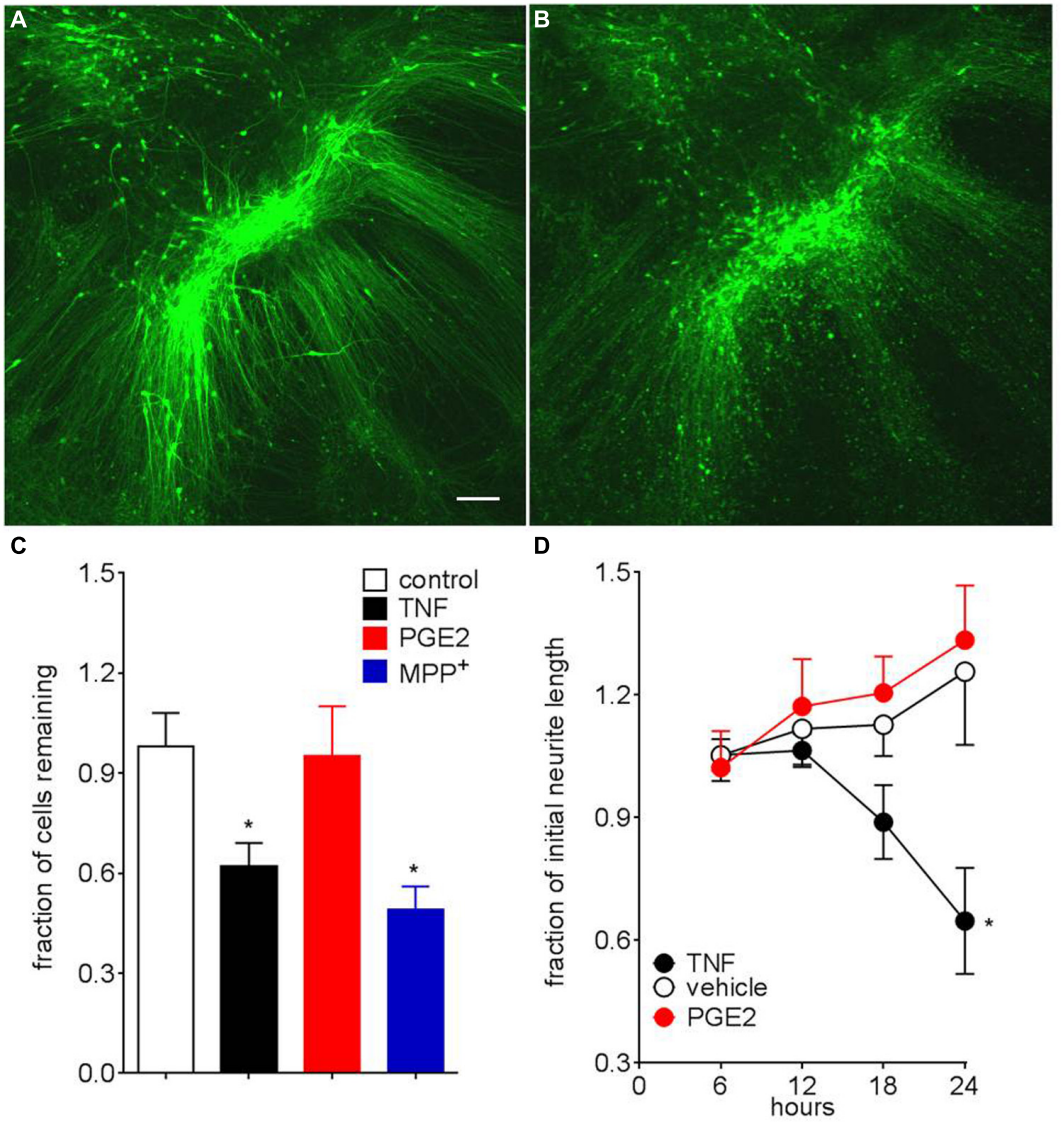
***PITX3^eGFP/w^* neuron death following MPP^+^ and TNF.** **(A)** Typical representative images of *PITX3^eGFP/w^* green neurons prior to **(A)** and after the addition of TNF (20 ng mL^-1^, 24 h, **B**). **(C)** Effects of MPP^+^ (5 μM) and tumor necrosis factor (20 ng mL^-1^), both 24 h on neurons survival (as defined by maintenance of somal fluorescence) (one way ANOVA with *post hoc* Dunnett’s test compared to vehicle, ^∗^ indicates *p* < 0.05, *n* = 3–12 differentiations). Prostaglandin E2 (300 nM) was without significant effect (not shown; **D**). Surviving neurons show neurite retraction following incubation with TNF (20 ng/ml), but not prostaglandin E2 (300 nM). Data presented as mean ± SEM (one way ANOVA with *post hoc* Dunnett’s test compared to vehicle, ^∗^ indicates *p* < 0.05, *n* = 6–12 differentiations). For death assays *n* = 6–12 fields of view. For fluorescent neurite tracing neurites length is expressed as a fraction of original length (i.e., pre-ligand) *n* = 4, with measurements taken from 16 neurons. Scale bar; 100 μm.

To establish *PITX3^eGFP/w^* neurons as a tool for modeling neurodegeneration in PD we tested the effects of the neurotoxin MPP^+^, as well as two neuroinflammatory mediators: TNF, as well as prostaglandin E_2_ [PGE_2_, which is elevated in response to MPP^+^ (Wang et al., 2005)] on mature *PITX3^eGFP/w^* neurons. **Figures 7A,B** show a typical field of view before (7A) and 24 h after TNF (20 ng mL^-1^, 7B). After 24-h exposure, MPP^+^ (5 μM) significantly (one-way ANOVA with *post hoc* Dunnett’s test, *p* < 0.05, *n* = 4 wells) reduced eGFP^+^ cell number by 49 ± 13%. TNF (20, but not 2 ng mL^-1^), PGE_2_ was without effect (**Figure 7C**, *n* = 10). Around 50% of cells were resistant to TNF, even when the incubation was continued for 72 h (not shown). When neurons did not clearly die we were able to measure GFP^+^ neurite lengths using Nikon software. Compared to vehicle control, TNF, but not PGE_2_ significantly (one-way ANOVA with *post hoc* Dunnett’s test, *p* < 0.05, *n* = 10 and 12) reduced neurite length over this time (**Figure 7D**). A lower concentration of TNF (2 ng mL^-1^ for 24 h) had no effect on neurite length (not shown).

## Discussion

Current mDA differentiation protocols yield heterogeneous neural cultures with mixed neurotransmitter and regional specifications. The first aim of this study was to generate a homologous hPSC reporter line to facilitate the identification, isolation, and characterization of mature mDA neurons. The second aim was to assess the responses of these neurons to pharmacological and toxicological stimuli. The endogenous *PITX3* promoter was chosen to drive eGFP since, in the adult CNS, PITX3 expression is restricted to DA neurons of the midbrain (Smidt et al., 1997, 2012), it also has a demonstrable role in the development, maturation, and survival of mDA neurons (Nunes et al., 2003). PITX3-driven eGFP could be seen as early as day 8 of differentiation, from day 20 to termination, cultures contained 10–15% intensely fluorescent and clearly identifiable *PITX3^eGFP/w^* neurons.

To investigate the transcriptional changes in the *PITX3^eGFP/w^* neurons we used flow cytometry to collect eGFP^+^ cells at days 20, 40, 60, and 80. Although flow cytometry became more difficult over time as cultures developed extensive neuronal processes, we were consistently able to collect around 8% live cells for total RNA extraction. The transcriptional profile of *PITX3^eGFP/w^* cells was indicative of an mDA phenotype, with both the midbrain and floor plate markers; *PITX3*, *EN1*, *PAX5*, *FOXA2*, *SHH,* and *CORIN* highly upregulated compared to eGFP^-^ cells. In addition, the eGFP^+^ population showed a down-regulation of the anterior and posterior CNS markers, *NKX2*.1, *FOXG1*, and *HOXA2.* In contrast, *LMX1A* and *WNT1* were, unexpectedly, relatively down-regulated at various time points in the eGFP^+^ fraction compared to the eGFP^-^ fraction. Although *LMX1A* and *WNT1* encode critical mediators of early dopaminergic specification (Chung et al., 2009), they are also involved in other neural differentiation programs; for example, *LMX1A* also directs early forebrain fate while *WNT1* is involved in neural crest differentiation (Sarnat and Flores-Sarnat, 2005). Thus we think it is likely that *LMX1A* and *WNT1* may be expressed in the eGFP^-^ population as part of these other programs. As the cultures developed it became clear that the midbrain and floor plate markers *FOXA2*, *SHH*, *CORIN*, and *PAX5* expression decreased in eGFP^+^ cells, most likely as a consequence of neuronal maturation (Matsushita et al., 2002). This idea was supported by our data showing the presence of *DAT* transcript after day 60, possibly indicating the development of synaptic interactions. Consistent with this idea, the synaptic marker *SYP* was highly upregulated in the eGFP^+^ fraction, indicating the likely formation of functional synapses (Wiedenmann and Franke, 1985). Our profiling data are therefore consistent with the idea that PITX3 expressing neurons continue to develop during maturation and terminal differentiation.

We then used immunocytochemistry to confirm the transcriptional profile of developing *PITX3^eGFP/w^* neurons. Thus, TH, FOXA2 and LMX1A immunoreactivity was present in most *PITX3^eGFP/w^* neurons. GIRK2 was not present in all *PITX3^eGFP/w^* neurons, however, possibly indicating subpopulations of mDA neurons (Thompson et al., 2005), or perhaps distinct stages in development. GABAergic immunoreactivity was observed during differentiation, however, it was rarely found in *PITX3^eGFP/w^* cells.

Functionally, the *PITX3^eGFP/w^* neurons displayed spontaneous elevations of [Ca^2+^]_i_ and were capable of spontaneous, GABA sensitive dopamine release. The variation in concentration of dopamine released during differentiation may be attributable to the state of maturity of the developing cultures: immature mDA neurons release dopamine to aid development through D_2_ autoreceptor-mediated Nurr1 activation (Kim et al., 2006), whereas mature neurons release dopamine predominantly through classic neurotransmitter release signaling (Snyder, 2011). These properties of *PITX3^eGFP/w^* neurons are largely consistent with our current understanding of mDA neuron function *in vivo* (Overton and Clark, 1997; Ji et al., 2009). To confirm neuron maturation in culture we tested *PITX3^eGFP/w^* neurons with live cell ion imaging studies as previously described (Lang et al., 2004; Raye et al., 2007; Khaira et al., 2011). As cultures matured we also saw both a decrease in [Cl^-^]_i_ as well as an increase in the proportion of *PITX3^eGFP/w^* neurons responding to GABA with increased [Cl^-^]_i_. These findings are consistent with a shift from immature to mature cultures (Rivera et al., 1999; Payne et al., 2003). As [Cl^-^]_i_ decreased, basal [Ca^2+^]_i_ increased across cultures, lending support to the idea that following the expression of PITX3, neurons continue a relatively slow process of functional maturation.

These *PITX3^eGFP/w^* neurons responded to ATP, NA, ACh, and Glut with elevations of [Ca^2+^]_i_, indicating they were functionally responsive to multiple neurotransmitters. As cultures developed it became evident that more neurons responded to a greater number of ligands. Mouse PSC-derived TH^+^ neurons demonstrate variability in their responses to different neurotransmitters, at a time when cultures are mature (Raye et al., 2007; Watmuff et al., 2012), however, the present results indicate that extended differentiation of hPSC-derived mDA neurons results in a more homogenous pharmacological response to these neurotransmitters. Although we have not further characterized the receptors present, our functional data are broadly consistent with reports of the presence of ionotropic and metabotropic glutamate, GABA, acetylcholine and ATP receptor transcripts in human substantia nigra (see for example, E-GEOD-7621 and E-GEOD-20333; http://www.ebi.ac.uk/). These tissues samples also reveal multiple α- and β- adrenergic receptors, a finding consistent with our evidence that noradrenaline is capable of elevating intracellular calcium in *PITX3^eGFP/w^* neurons.

VOCCs are present on mDA neurons *in vivo* (Catterall, 2011) and their tightly controlled activation contributes to the spontaneous activity observed in rodent dopaminergic neurons (see Marinelli et al., 2006). Our observation of spontaneous oscillations of intracellular calcium is likely to result from bursts of spontaneous electrical activity, clearly these events require extensive electrophysiological characterization before conclusions about their significance can be drawn. We also showed that a high concentration of nifedipine and as well as mibefradil, but not MVIIA reduced the elevation in [Ca^2+^]_i_ following KCl depolarization, indicating a role for L- and T- type VOCCs in hPSC-derived neuronal Ca^2+^ signaling. While we acknowledge that the single concentrations of inhibitor used in this study are not optimal we argue that the effects we see most likely indicate that these channels are present and functional in these cultures. Clearly, there is a need for further electrophysiological studies to pursue the identity and function of these channels. In addition, our studies are somewhat spatially limited in that they account only for Ca^2+^ channel present in the soma and they in no way allow prediction of which channel subunits may be present on the soma vs. dendrites; it is for example, dendritic calcium that regulates spontaneous activity, at least in dissociated rat neurons (Kim et al., 2013). It is evident from array data (see E-GEOD-7621 and E-GEOD-20333; http://www.ebi.ac.uk/) that multiple voltage gated channel subunits may be present in the human substantia nigra, but how these channels cooperate to regulate spontaneous bursts of activity is unclear.

Intracellular chloride decreases as neurons mature, a phenomenon that corresponds to a GABA related depolarization of immature neurons (Ben-Ari et al., 2007). Our cultured neurons showed a significant decrease in intracellular chloride over differentiation which correlates with a change in response to GABA where, early in differentiation GABA was a depolarizing agent, capable of reducing intracellular chloride, later in differentiation it increased intracellular chloride and inhibited dopamine release. Together these finding indicate a developing neuronal culture that largely consists of mature neurons from day 60 of differentiation. This last point is an important consideration as it demonstrates the importance of choosing an appropriate time for experimentation or implantation, although the fundamental significance of this work remains that *PITX3^eGFP/w^* neurons are most useful for neuropharmacological or neurophysiological studies (albeit from at least 60 days in culture).

In our next series of experiments we investigated the effects of the neurotoxin MPP^+^ and two neuroinflammatory mediators, TNF and PGE2 on *PITX3^eGFP/w^* neuron survival and neurite retraction. We chose these ligands since TNF is elevated in the striatal tissue of mice and rats following the injection of the neurotoxins, MPTP or 6-hydroxydopamine (Mogi et al., 1999; Sriram et al., 2002), while mice lacking TNF receptors are insensitive to the effects of MPTP (Sriram et al., 2002). Furthermore the TNF inhibitor protein, dominant negative TNF, protects substantia nigral dopaminergic neurons against 6-hydroxydopamine-induced neuronal toxicity (McCoy et al., 2006; Harms et al., 2011). Thus evidence linking TNF to the initiation and progression of PD is strong. In contrast to TNF, PGE_2_ has four cognate receptor subtypes, EPR1-4. Carrasco et al. (2007) have found that EPR2 activation protects mDA neurons against low levels of oxidative stress while EPR1 activation renders mDA neurons vulnerable to such stress. A 24 h exposure to TNF, but not PGE_2_ significantly reduced the number of eGFP fluorescent cell bodies in culture and also induced the retraction of GFP^+^ neurites. Death of neurons occurred within 16 h of TNF addition, but neurite retraction was maximal at 24 h. Significantly, our data indicates that not all *PITX3^eGFP/w^* neurons were sensitive to the effects of TNF possibly indicating the presence of subtypes of *PITX3^eGFP/w^* neurons. Whether this difference equates to the difference between nigral and ventral tegmental dopaminergic neurons, or reflects the largely unknown identities of other cells in each well, is currently unknown.

As a final step we assessed *PITX3^eGFP/w^* neuron survival following the addition of the neurotoxin, MPP^+^. MPTP, the precursor of MPP^+^ is a well-described dopaminergic neurotoxin that is likely to produce excessive levels of reactive oxygen species and or an inhibition of mitochondrial activity to kill neurons (Smeyne and Jackson-Lewis, 2005). In this study a 24 h exposure to MPP^+^ significantly reduced eGFP fluorescence, a finding consistent with its reported activities* in vivo* and *in vitro*. Of significance is a report by Luk et al. (2013) who suggest that the absence of one allele of Pitx3 in a mouse system increases sensitivity of TH^+^ neurons to MPTP. This work may indicate some potential for *PITX3* haploinsufficiency to increase the sensitivity of these PITX3^+^ neurons to neurotoxin-mediated death. However, we would argue that, in the absence of neurotoxic insult, these neurons are readily maintained in culture for at least 80 days. We interpret this as evidence that sufficient *PITX3* is present to maintain survival.

## Conclusion

We believe that progress in determining factor(s) that initiate and sustain idiopathic PD is hampered by the lack of a suitable *in vitro* model of human mDA neuron function. The hPSC *PITX3^eGFP/w^* reporter cell line described here has the potential to accelerate advancement in neurobiology, neuropharmacology, neurophysiology, and neurotoxicology as it permits the identification, sorting and tracking of live and dying mDA neurons in culture. These *PITX3^eGFP/w^* neurons show both receptor expression and activities that appear consistent with animal models and, more importantly, human array data. In particular we believe that these *PITX3^eGFP/w^* neurons will become a powerful tool in drug discovery biology as they readily permit investigation of the mechanisms underlying neuron function and degeneration.

## Conflict of Interest Statement

The authors declare that the research was conducted in the absence of any commercial or financial relationships that could be construed as a potential conflict of interest.

## References

[B1] Ben-Ari Y. Gaiarsa J. L. Tyzio R. Khazipov R. (2007). GABA: a pioneer transmitter that excites immature neurons and generates primitive oscillations. *Physiol. Rev.* 87 1215–1284 10.1152/physrev.00017.2006 17928584

[B2] Bialecka M. Klodowska-Duda G. Kurzawski M. Slawek J. Gorzkowska A. Opala G. (2008). Interleukin-10 (IL10) and tumor necrosis factor alpha (TNF) gene polymorphisms in Parkinson’s disease patients. *Parkinsonism Relat. Disord.* 14 636–640 10.1016/j.parkreldis.2008.02.001 18362084

[B3] Brewer G. J. Torricelli J. R. Evege E. K. Price P. J. (1993). Optimized survival of hippocampal neurons in B27-supplemented neurobasal, a new serum-free medium combination. *J. Neurosci. Res.* 35 567–576 10.1002/jnr.490350513 8377226

[B4] Carrasco E. Casper D. Werner P. (2007). PGE(2) receptor EP1 renders dopaminergic neurons selectively vulnerable to low-level oxidative stress and direct PGE(2) neurotoxicity. *J. Neurosci. Res.* 85 3109–3117 10.1002/jnr.21425 17868147

[B5] Catterall W. A. (2011). Voltage-gated calcium channels. *Cold Spring Harb. Perspect. Biol.* 3:a003947 10.1101/cshperspect.a003947 PMC3140680 21746798

[B6] Chung S. Leung A. Han B. S. Chang M. Y. Moon J. I. Kim C. H. (2009). Wnt1-lmx1a forms a novel autoregulatory loop and controls midbrain dopaminergic differentiation synergistically with the SHH-FoxA2 pathway. *Cell Stem Cell* 5 646–658 10.1016/j.stem.2009.09.015 19951692 PMC2788512

[B7] Devine M. J. Ryten M. Vodicka P. Thomson A. J. Burdon T. Houlden H. (2011). Parkinson’s disease induced pluripotent stem cells with triplication of the alpha-synuclein locus. *Nat. Commun.* 2 440 10.1038/ncomms1453 PMC3265381 21863007

[B8] Gerhard A. Pavese N. Hotton G. Turkheimer F. Es M. Hammers A. (2006). In vivo imaging of microglial activation with [11C](R)-PK11195 PET in idiopathic Parkinson’s disease. *Neurobiol. Dis.* 21 404–412 10.1016/j.nbd.2005.08.002 16182554

[B9] Groden D. L. Guan Z. Stokes B. T. (1991). Determination of Fura-2 dissociation constants following adjustment of the apparent Ca-EGTA association constant for temperature and ionic strength. *Cell Calcium* 12 279–287 10.1016/0143-4160(91)90002-V 1906783

[B10] Grynkiewicz G. Poenie M. Tsien R. Y. (1985). A new generation of Ca^2+^ indicators with greatly improved fluorescence properties. *J. Biol. Chem.* 260 3440–3450. 3838314

[B11] Harms A. S. Barnum C. J. Ruhn K. A. Varghese S. Trevino I. Blesch A. (2011). Delayed dominant-negative TNF gene therapy halts progressive loss of nigral dopaminergic neurons in a rat model of Parkinson’s disease. *Mol. Ther.* 19 46–52 10.1038/mt.2010.217 20959812 PMC3017447

[B12] Hartley B. J. Fabb S. A. Finnin B. A. Haynes J. M. Pouton C. W. (2014). Zinc-finger nuclease enhanced gene targeting in human embryonic stem cells. *J. Vis. Exp.* 2014:e51764 10.3791/51764 PMC4758769 25177806

[B13] Haynes J. M. Iannazzo L. Majewski H. (2002). Phorbol ester-induced contractility and Ca^2+^ influx in human cultured prostatic stromal cells. *Biochem. Pharmacol.* 64 385–392 10.1016/S0006-2952(02)01211-X 12147289

[B14] Hockemeyer D. Soldner F. Beard C. Gao Q. Mitalipova M. DeKelver R. C. (2009). Efficient targeting of expressed and silent genes in human ESCs and iPSCs using zinc-finger nucleases. *Nat. Biotechnol.* 27 851–857 10.1038/nbt.1562 19680244 PMC4142824

[B15] Inglefield J. R. Schwartz-Bloom R. D. (1999). Fluorescence imaging of changes in intracellular chloride in living brain slices. *Methods* 18 197–203 10.1006/meth.1999.0772 10356351

[B16] Ji H. Hougaard C. Herrik K. F. Strobaek D. Christophersen P. Shepard P. D. (2009). Tuning the excitability of midbrain dopamine neurons by modulating the Ca^2+^ sensitivity of SK channels. *Eur. J. Neurosci.* 29 1883–1895 10.1111/j.1460-9568.2009.06735.x 19473240 PMC4430859

[B17] Khaira S. K. Nefzger C. M. Beh S. J. Pouton C. W. Haynes J. M. (2011). Midbrain and forebrain patterning delivers immunocytochemically and functionally similar populations of neuropeptide Y containing GABAergic neurons. *Neurochem. Int.* 59 413–420 10.1016/j.neuint.2011.02.016 21349310

[B18] Khaira S. K. Pouton C. W. Haynes J. M. (2009). P2X2 P2X4 and P2Y1 receptors elevate intracellular Ca^2+^ in mouse embryonic stem cell-derived GABAergic neurons. *Br. J. Pharmacol.* 158 1922–1931 10.1111/j.1476-5381.2009.00479.x 20050186 PMC2807654

[B19] Kim J. H. Auerbach J. M. Rodriguez-Gomez J. A. Velasco I. Gavin D. Lumelsky N. (2002). Dopamine neurons derived from embryonic stem cells function in an animal model of Parkinson’s disease. *Nature* 418 50–56 10.1038/nature00900 12077607

[B20] Kim S. Y. Choi K. C. Chang M. S. Kim M. H. Kim S. Y. Na Y. S. (2006). The dopamine D2 receptor regulates the development of dopaminergic neurons via extracellular signal-regulated kinase and Nurr1 activation. *J. Neurosci*. 26 4567–4576 10.1523/JNEUROSCI.5236-05.2006 16641236 PMC6674082

[B21] Kim S. H. Jang J. Y. Jang M. Um K. B. Chung S. Kim H. J. (2013). Homeostatic regulation mechanism of spontaneous firing determines glutamate responsiveness in the midbrain dopamine neurons. *Cell Calcium* 54 295–306 10.1016/j.ceca.2013.07.004 23988034

[B22] Krapf R. Berry C. A. Verkman A. S. (1988). Estimation of intracellular chloride activity in isolated perfused rabbit proximal convoluted tubules using a fluorescent indicator. *Biophys. J.* 53 955–962 10.1016/S0006-3495(88)83176-X 3395662 PMC1330276

[B23] Kriks S. Shim J. W. Piao J. Ganat Y. M. Wakeman D. R. Xie Z. (2011). Dopamine neurons derived from human ES cells efficiently engraft in animal models of Parkinson’s disease. *Nature* 480 547–571 10.1038/nature10648 22056989 PMC3245796

[B24] Lang R. J. Haynes J. M. Kelly J. Johnson J. Greenhalgh J. O’Brien C. (2004). Electrical and neurotransmitter activity of mature neurons derived from mouse embryonic stem cells by Sox-1 lineage selection and directed differentiation. *Eur. J. Neurosci.* 20 3209–3221 10.1111/j.1460-9568.2004.03782.x 15610154

[B25] Lindvall O. Kokaia Z. Martinez-Serrano A. (2004). Stem cell therapy for human neurodegenerative disorders-how to make it work. *Nat. Med. 10(Suppl.),* S42–S50 10.1038/nm1064 15272269

[B26] Livak K. J. Schmittgen T. D. (2001). Analysis of relative gene expression data using real-time quantitative PCR and the 2(-Delta Delta C(T)) method. *Methods* 25 402–408 10.1006/meth.2001.1262 11846609

[B27] Luk K. C. Rymar V. V. van den Munckhof P. Nicolau S. Steriade C. Bifsha P. (2013). The transcription factor Pitx3 is expressed selectively in midbrain dopaminergic neurons susceptible to neurodegenerative stress. *J. Neurochem.* 125 932–943 10.1111/jnc.12160 23331067

[B28] Marinelli M. Rudick C. N. Hu X. T. White F. J. (2006). Excitability of dopamine neurons: modulation and physiological consequences. *CNS Neurol. Disord. Drug Targets* 5 79–97 10.2174/187152706784111542 16613555

[B29] Matsushita N. Okada H. Yasoshima Y. Takahashi K. Kiuchi K. Kobayashi K. (2002). Dynamics of tyrosine hydroxylase promoter activity during midbrain dopaminergic neuron development. *J. Neurochem.* 82 295–304 10.1046/j.1471-4159.2002.00972.x 12124430

[B30] McCoy M. K. Martinez T. N. Ruhn K. A. Szymkowski D. E. Smith C. G. Botterman B. R. (2006). Blocking soluble tumor necrosis factor signaling with dominant-negative tumor necrosis factor inhibitor attenuates loss of dopaminergic neurons in models of Parkinson’s disease. *J. Neurosci.* 26 9365–9375 10.1523/JNEUROSCI.1504-06.2006 16971520 PMC3707118

[B31] McCoy M. K. Ruhn K. A. Martinez T. N. McAlpine F. E. Blesch A. Tansey M. G. (2008). Intranigral lentiviral delivery of dominant-negative TNF attenuates neurodegeneration and behavioral deficits in hemiparkinsonian rats. *Mol. Ther.* 16 1572–1579 10.1038/mt.2008.146 18628756 PMC2670754

[B32] Mogi M. Harada M. Riederer P. Narabayashi H. Fujita K. Nagatsu T. (1994). Tumor necrosis factor-alpha (TNF-alpha) increases both in the brain and in the cerebrospinal fluid from parkinsonian patients. *Neurosci. Lett.* 165 208–210 10.1016/0304-3940(94)90746-3 8015728

[B33] Mogi M. Togari A. Kondo T. Mizuno Y. Komure O. Kuno S. (2000). Caspase activities and tumor necrosis factor receptor R1 (p55) level are elevated in the substantia nigra from parkinsonian brain. *J. Neural. Transm.* 107 335–341 10.1007/s007020050028 10821442

[B34] Mogi M. Togari A. Tanaka K. Ogawa N. Ichinose H. Nagatsu T. (1999). Increase in level of tumor necrosis factor (TNF)-alpha in 6-hydroxydopamine-lesioned striatum in rats without influence of systemic L-DOPA on the TNF-alpha induction. *Neurosci. Lett.* 268 101–104 10.1016/S0304-3940(99)00388-2 10400088

[B35] Nagatsu T. Sawada M. (2007). Biochemistry of postmortem brains in Parkinson’s disease: historical overview and future prospects. *J. Neural Transm. Suppl.* 2007 113–120 10.1007/978-3-211-73574-9_14 17982884

[B36] Nefzger C. M. Su C. T. Fabb S. A. Hartley B. J. Beh S. J. Zeng W. R. (2012). Lmx1a allows context-specific isolation of progenitors of GABAergic or dopaminergic neurons during neural differentiation of embryonic stem cells. *Stem Cells* 30 1349–1361 10.1002/stem.1105 22495882

[B37] Nguyen H. N. Byers B. Cord B. Shcheglovitov A. Byrne J. Gujar P. (2011). LRRK2 mutant iPSC-derived DA neurons demonstrate increased susceptibility to oxidative stress. *Cell Stem Cell* 8 267–280 10.1016/j.stem.2011.01.013 21362567 PMC3578553

[B38] Nishimura M. Mizuta I. Mizuta E. Yamasaki S. Ohta M. Kaji R. (2001). Tumor necrosis factor gene polymorphisms in patients with sporadic Parkinson’s disease. *Neurosci. Lett.* 311 1–4 10.1016/S0304-3940(01)02111-5 11585553

[B39] Nunes I. Tovmasian L. T. Silva R. M. Burke R. E. Goff S. P. (2003). Pitx3 is required for development of substantia nigra dopaminergic neurons. *Proc. Natl. Acad. Sci. U.S.A.* 100 4245–4250 10.1073/pnas.0230529100 12655058 PMC153078

[B40] Overton P. G. Clark D. (1997). Burst firing in midbrain dopaminergic neurons. *Brain Res. Brain Res. Rev.* 25 312–334 10.1016/S0165-0173(97)00039-8 9495561

[B41] Payne J. A. Rivera C. Voipio J. Kaila K. (2003). Cation-chloride co-transporters in neuronal communication, development and trauma. *Trends Neurosci.* 26 199–206 10.1016/S0166-2236(03)00068-7 12689771

[B42] Raye W. S. Tochon-Danguy N. Pouton C. W. Haynes J. M. (2007). Heterogeneous population of dopaminergic neurons derived from mouse embryonic stem cells: preliminary phenotyping based on receptor expression and function. *Eur. J. Neurosci.* 25 1961–1970 10.1111/j.1460-9568.2007.05489.x 17419751

[B43] Rivera C. Voipio J. Payne J. A. Ruusuvuori E. Lahtinen H. Lamsa K. (1999). The K^+^/Cl^-^ co-transporter KCC2 renders GABA hyperpolarizing during neuronal maturation. *Nature* 397 251–255 10.1038/16697 9930699

[B44] Sanchez-Danes A. Consiglio A. Richaud Y. Rodriguez-Piza I. Dehay B. Edel M. (2012). Efficient generation of A9 midbrain dopaminergic neurons by lentiviral delivery of LMX1A in human embryonic stem cells and induced pluripotent stem cells. *Hum. Gene Ther.* 23 56–69 10.1089/hum.2011.054 21877920 PMC3472681

[B45] Sarnat H. B. Flores-Sarnat L. (2005). Embryology of the neural crest: its inductive role in the neurocutaneous syndromes. *J. Child Neurol.* 20 637–643 10.1177/08830738050200080101 16225807

[B46] Smeyne R. J. Jackson-Lewis V. (2005). The MPTP model of Parkinson’s disease. *Brain Res. Mol. Brain Res.* 134 57–66 10.1016/j.molbrainres.2004.09.017 15790530

[B47] Smidt M. P. van Schaick H. S. Lanctot C. Tremblay J. J. Cox J. J. van der Kleij A. A. (1997). A homeodomain gene Ptx3 has highly restricted brain expression in mesencephalic dopaminergic neurons. *Proc. Natl. Acad. Sci. U.S.A.* 94 13305–13310 10.1073/pnas.94.24.13305 9371841 PMC24304

[B48] Smidt M. P. von Oerthel L. Hoekstra E. J. Schellevis R. D. Hoekman M. F. (2012). Spatial and temporal lineage analysis of a Pitx3-driven Cre-recombinase knock-in mouse model. *PLoS ONE* 7:e42641 10.1371/journal.pone.0042641 PMC3411649 22870339

[B49] Snyder S. H. (2011). What dopamine does in the brain. *Proc. Natl. Acad. Sci. U.S.A.* 108 18869–18871 10.1073/pnas.1114346108 22106252 PMC3223473

[B50] Sriram K. Matheson J. M. Benkovic S. A. Miller D. B. Luster M. I. O’Callaghan J. P. (2002). Mice deficient in TNF receptors are protected against dopaminergic neurotoxicity: implications for Parkinson’s disease. *FASEB J.* 16 1474–1476. 12205053 10.1096/fj.02-0216fje

[B51] Sriram K. Matheson J. M. Benkovic S. A. Miller D. B. Luster M. I. O’Callaghan J. P. (2006). Deficiency of TNF receptors suppresses microglial activation and alters the susceptibility of brain regions to MPTP-induced neurotoxicity: role of TNF-alpha. *FASEB J.* 20 670–682 10.1096/fj.05-5106com 16581975

[B52] Thompson L. Barraud P. Andersson E. Kirik D. Bjorklund A. (2005). Identification of dopaminergic neurons of nigral and ventral tegmental area subtypes in grafts of fetal ventral mesencephalon based on cell morphology, protein expression, and efferent projections. *J. Neurosci.* 25 6467–6477 10.1523/JNEUROSCI.1676-05.2005 16000637 PMC6725273

[B53] Wang T. Pei Z. Zhang W. Liu B. Langenbach R. Lee C. (2005). MPP^+^-induced COX-2 activation and subsequent dopaminergic neurodegeneration. *FASEB J.* 19 1134–1136. 15845609 10.1096/fj.04-2457fje

[B54] Watmuff B. Pouton C. W. Haynes J. M. (2012). In vitro maturation of dopaminergic neurons derived from mouse embryonic stem cells: implications for transplantation. *PLoS ONE* 7:e31999 10.1371/journal.pone.0031999 PMC3285205 22384125

[B55] Wiedenmann B. Franke W. W. (1985). Identification and localization of synaptophysin, an integral membrane glycoprotein of Mr 38000 characteristic of presynaptic vesicles. *Cell* 41 1017–1028 10.1016/S0092-8674(85)80082-9 3924408

